# Dr. Elon G. Douglas

**Published:** 1913-05-15

**Authors:** 


Dr. Elon G. Douglas. Dr. Douglas for many years a well-known practitioner of Lapeer, Mich., died at the age of 74 at his home. Dr. Douglas was an old school gentleman and a man of high professional ideals and of the strictest integrity. While he was never a great contributor to professional work he was a frequent attendant of the state and local society meetings.

